# Parametric Studies and Semi-Continuous Harvesting Strategies for Enhancing CO_2_ Bio-Fixation Rate and High-Density Biomass Production Using Adaptive Laboratory-Evolved *Chlorella vulgaris*

**DOI:** 10.3390/microorganisms14020324

**Published:** 2026-01-30

**Authors:** Sufia Hena, Tejas Bhatelia, Nadia Leinecker, Milinkumar Shah

**Affiliations:** Western Australia School of Mines: Minerals, Energy and Chemical Engineering, Curtin University, Perth, WA 6102, Australia; t.bhatelia@curtin.edu.au (T.B.); nadia.leinecker@curtin.edu.au (N.L.); milinkumar.shah@curtin.edu.au (M.S.)

**Keywords:** process optimisation, parametric effects, CO_2_ sequestration, biomass productivity, green technologies

## Abstract

This study adopts a biochemical approach to sequester CO_2_ while producing biomass rich in protein and lipids, using an adapted strain of *Chlorella vulgaris* (ALE-*Cv*), which had previously evolved to tolerate a gas mixture containing 10% CO_2_ and 90% air. The research studied the operating parameters of the batch photobioreactor for ALE-*Cv* to evaluate the effects of inoculum size, photoperiod, light intensity, pH of culture, and CO_2_ supply rate on biomass productivity and CO_2_ bio-fixation rate. The optimal conditions were identified as 16:8 h light–dark cycles, 5000 lux, pH 7, 20 mL of 10 g/L inoculum, and 0.6 VVM; the system achieved a maximum total biomass production of 7.03 ± 0.21 g/L with a specific growth rate of 0.712 day^−1^, corresponding to a CO_2_ bio-fixation of 13.4 ± 0.45 g/L in batch cultivation. While the pre-adapted strain of *Chlorella vulgaris* under the same operating conditions, except for the gas supply, which was air, achieved a maximum total biomass production of 0.52 ± 0.008 g/L, and the total CO_2_ bio-fixation was 1.036 ± 0.021 g/L during 7-day cultivation. A novel semi-continuous harvesting process, with and without nutrient addition, was also investigated to maximise biomass yield and enable water recycling for culture media. The maximum biomass production in semi-continuous harvesting process with and without nutrition added was 5.29 ± 0.09 and 9.91 ± 0.11 g/L, while the total corresponding CO_2_ bio-fixation was 9.70 ± 0.13 and 18.16 ± 0.11 g/L, respectively, during 15-day cultivation. The findings provide critical insights into enhancing CO_2_ bio-fixation through adaptive evolution of ALE-*Cv* and offer optimal operational parameters for future large-scale microalgae cultivation. This research also links microalgae-based CO_2_ sequestration to green technologies and the bioeconomy, highlighting its potential contribution to climate change mitigation while supporting environmental sustainability, food security, and ecosystem resilience.

## 1. Introduction

The increasing global demand for renewable energy, sustainable food sources, and environmental remediation technologies has positioned microalgae as a keystone of the emerging bioeconomy [1]. Microalgae are microscopic photosynthetic organisms capable of converting carbon dioxide (CO_2_), water, and sunlight into biomass rich in lipids, carbohydrates, and proteins [2]. These organisms fix CO_2_ through oxygenic photosynthesis with efficiencies exceeding those of terrestrial plants, capturing up to 1.8 kg of CO_2_ per kilogram of dry biomass, which makes them powerful candidates for carbon capture and utilisation (CCU) strategies aimed at reducing atmospheric CO_2_ levels [3].

Their extraordinary productivity, versatility, and capacity to thrive in diverse aquatic environments make microalgae promising feedstocks for biofuels, bioplastics, animal feed, and nutraceuticals [4]. However, despite their biotechnological potential, the high cost of microalgal biomass production remains a major barrier to commercialisation [5]. Increasing cell density during cultivation is considered one of the most effective strategies to reduce production costs, as it enhances volumetric productivity while minimising resource inputs, energy demand, and downstream processing requirements [6]. Higher cell densities also enhance CO_2_ uptake per unit area, providing additional climate mitigation benefits compared to terrestrial carbon sinks, which require extensive land areas and store carbon more slowly [7]. Nevertheless, dense microalgal cultures introduce biophysical and physiological constraints, including self-shading that limits light penetration, oxygen supersaturation, and accumulation of inhibitory metabolites [8,9]. Overcoming these limitations requires optimised mixing, light management, and CO_2_ delivery systems [10].

Efforts to increase cell density encompass both biological and process-engineering strategies. Biologically, strain selection, adaptive laboratory evolution, and genetic modification are used to maintain high productivity under dense culture conditions. Vani et al. (2023) generated mutants of *C. reinhardtii* with shortened light-harvesting antennae; the biomass of the mutants was significantly higher by 12% in CRCM7 and 34% in CRCM13 than in wild-type *Chlamydomonas reinhardtii* (cc125) [11]. Earlier, Shin et al. (2016) improved the photosynthetic efficiency of *Chlorella vulgaris* by producing reduced chlorophyll antenna size using ethyl methanesulfonate. The mutant cultures achieved 44.5% improvement in biomass productivity under high light (200 μmol photons m^−2^ s^−1^) [12].

Process optimisation also plays a critical role in enhancing cell density. Light intensity, photoperiod, pH, and inoculum size are among the most influential parameters [13,14,15,16,17,18,19]. Light distribution becomes increasingly heterogeneous in dense cultures, causing excess light exposure near the surface and light limitation deeper in the culture. This light gradient reduces overall photosynthetic efficiency. Previous studies have demonstrated that appropriate light intensities and photoperiods significantly enhance microalgal biomass yield. For instance, Hena et al. [13] reported that increasing light intensity from 160 to 200 μmol m^−2^ s^−1^ increased biomass concentration from 4 g/L to 5 g/L. Wahidin et al. [14] observed improved growth of *Nannochloropsis* sp. at 100 μmol m^−2^ s^−1^ compared to 50 μmol m^−2^ s^−1^, and that an 18 h light: 6 h dark photoperiod increased specific growth rate. Optimal photoperiods for microalgal cultivation typically range from 12:12 to 16:08 h [15]. Light intensities above saturation induce photoinhibition, which can be mitigated by alternating cycles of light and darkness [16]. Culture pH also affects CO_2_ availability, as it determines the balance between dissolved CO_2_ and bicarbonate (HCO_3_^−^). Although microalgae tolerate a wide pH range, growth is maximised near species-specific optima [17], and maintaining appropriate pH significantly improves CO_2_ capture efficiency [18,19]. Efficient hydrodynamics and mixing are equally essential for redistributing cells across illuminated zones and improving CO_2_ mass transfer, particularly at high biomass concentrations [10,20]. Helisch et al. (2020) conducted an interesting study for future bioregenerative life support in space, culturing *Chlorella vulgaris* in a novel microgravity-capable membrane raceway photobioreactor and achieved 12.2 g/L biomass with a maximum productivity of 1.3 g/L/day [21].

The mode of cultivation further influences cell density and productivity. Fed-batch and semi-continuous harvesting strategies help sustain exponential growth by supplying nutrients and CO_2_ incrementally and avoiding nutrient depletion [22,23]. The fed-batch cultivation is widely adopted in industrial applications, as it prolongs the culture’s productive phase and consistently achieves elevated microalgal biomass concentrations [23].

In semi-continuous harvesting, a portion of the culture is periodically replaced with fresh medium, extending the rapid-growth phase, improving biomass yield and CO_2_ capture, and reducing the accumulation of extracellular polymeric substances (EPS), which otherwise hinder light penetration [24]. However, it is crucial to optimise the intervals of harvesting culture and replacing it with fresh culture media. Aowtrakool et al. [25] reported enhanced production of a water-soluble phycocyanin, a blue photosynthetic pigment from *Arthrospira platensis*, under a semi-continuous regime.

Despite extensive research efforts, the cost of microalgae cultivation remains high, primarily due to excessive energy and water consumption. In this study, biomass productivity was enhanced using an adapted strain of *Chlorella vulgaris* capable of growth under elevated CO_2_ concentrations (10%), resulting in higher biomass productivity compared to the wild-type strain. To the best of our knowledge, no previous studies have reported the simultaneous enhancement of biomass productivity and culture medium recycling using an adaptive laboratory-evolved strain of *Chlorella vulgaris*. The semi-continuous harvesting approach applied to adaptive laboratory-evolved *Chlorella vulgaris* (ALE-*Cv*) offers significant advantages for advancing green technologies and the bioeconomy by simultaneously converting CO_2_ into biomass and reducing water consumption in microalgal cultivation systems, emphasising its potential to contribute to climate change mitigation while supporting environmental sustainability, food security, and ecosystem resilience.

## 2. Materials and Methods

### 2.1. Materials (Culture Media and Microalgal Strain)

The microalga *Chlorella vulgaris* (ALE-*Cv*) used in this study was previously adapted in the laboratory at 10% CO_2_ mixed with 90% air (*v*/*v*) using *Chlorella vulgaris* CS41 purchased from CSIRO, Hobart, Australia. During the adaptation process, the CO_2_ concentration in the gas stream gradually increased from 0.03 to 10 vol %. The adaptation was completed in almost 80 days. The detailed adaptation method of *Chlorella vulgaris* CS41 is described elsewhere [26]. After the adaptation, the strain was named ALE-*Cv*. ALE-*Cv* was cultivated in commercial BG11 culture medium (PhytoTechnology Laboratories, Lenexa, KS, USA), in which the sodium nitrate (Thermo Fisher Scientific, Melbourne, Australia) concentration was adjusted to 4.5 g/L; all other components were unchanged. All chemicals used in the study were of analytical grade.

### 2.2. ALE-Cv Culture, Growth, and Maintenance

The culture media was prepared by dissolving 1.68 g of commercial BG11 and 3 g of sodium nitrate in 1 L of Milli-Q water. After complete dissolution, the pH of the culture media was adjusted to 7 using 0.1 M aqueous NaOH, followed by autoclaving at 121 °C for 20 min at 16 atm. Freshly prepared BG11 culture medium was inoculated with 8 mL of ALE-*Cv* culture (0.75 g/L; 2% *v*/*v*) to achieve an initial biomass concentration of 0.015 g/L dry weight in a 1 L photobioreactor with a 400 mL working volume. The bioreactors were placed inside a semi-automatic incubator with temperature and LED light source controls. All bioreactors were supplied with a gas mixture of 10% CO_2_ and 90% air (*v*/*v*) using flow control valves to maintain a defined volumetric flow rate per unit volume of culture medium (VVM) for 7 days. Biomass productivity, specific growth rate, and CO_2_ bio-fixation were determined as described below. Chlorophyll-a extraction and quantification were performed following the method reported by Hena et al. [27].

### 2.3. Study of ALE-Cv Growth Parameters

The ranges of the process parameters investigated are summarised in Table 1. The experiments were conducted following the sequence outlined in Table 1, as described in our previous study [26]. Operating conditions were systematically evaluated to identify the settings that maximised biomass productivity, specific growth rate, and CO_2_ bio-fixation. All experiments were performed in triplicate to ensure reproducibility.

#### 2.3.1. Study the Effect of Photoperiod

The growth of ALE-*Cv* in BG11 medium was monitored under different photoperiods (light:dark; 12 h:12 h; 16 h:8 h, 18 h:6 h, 20 h:4 h, and 24 h:0 h), while all other operating parameters were maintained as specified in Table 1. Light and dark cycles were kept constant for over 24 h for each experiment.

#### 2.3.2. Study the Effect of Light Intensity

The effect of light intensity on ALE-*Cv* biomass growth was investigated while keeping the photoperiod (light:dark; 16 h:8 h) constant. Light intensity varied from 3000 to 5500 lux, as indicated in Table 1.

#### 2.3.3. Study the Effect of pH

The effect of culture pH on ALE-*Cv* biomass growth was examined within a range of 6 to 9 (Table 1), while maintaining the light intensity and photoperiod based on optimal conditions identified in earlier experiments. The pH of the cultures was measured and adjusted twice daily: once 30 min after the start of the light period, and once 30 min after the onset of the dark period. The pH was maintained within ±0.05 of the target using 0.1 M NaOH or 0.1 M HCl.

#### 2.3.4. Study the Effect of Inoculum Size

The effect of inoculum size on ALE-C*v* biomass growth and CO_2_ bio-fixation rates was investigated using two experimental approaches, as outlined in Table 1:

(i) Four different biomass concentrations (g/L) were tested while keeping the inoculum volume constant.
(ii) Three different inoculum volumes (1%, 5%, and 10% of the total culture) were evaluated using the same biomass concentration (g/L).

#### 2.3.5. Study the Effect of VVM Aided with and Without a Magnetic Stirrer

A 20 mL inoculum of ALE-*Cv* (10 g/L) was used to evaluate the effect of different volumetric gas flow rates (VVM) on biomass productivity and CO_2_ bio-fixation. The tested VVM range was 1.0–0.5, as listed in Table 1. To further assess the impact of culture mixing at lower aeration rates, additional experiments were conducted at 0.5 and 0.3 VVM using a magnetic stirrer operating at 100 rpm. The performance of cultures grown under these conditions was compared with those operated at the same VVM values without stirring.

The magnetic stirrer was introduced to enhance biomass suspension and gas–liquid mass transfer, since visible biomass settling and reduced growth were observed at lower aeration rates in non-stirred cultures.

### 2.4. Semi-Continuous Harvesting

The semi-continuous harvesting process was performed at a gas supply rate of 0.6 VVM using an initial biomass concentration of 0.44 g/L to maximise biomass growth within the shortest possible cultivation time. Two harvesting strategies were evaluated:

(i) Media replacement approach:

On day 7, 50% of the culture volume was harvested and immediately replenished with fresh BG11 medium to restore the working volume of the photobioreactor.

(ii) Spent media reuse approach:

On day 7, 50% of the culture was centrifuged at 4700 rpm for 10 min at room temperature. The biomass pellet was removed, and the recovered supernatant was filtered through a sterile 0.2 µm vacuum filter to prevent contamination before being returned to the bioreactor without the addition of fresh medium or nutrients.

### 2.5. Instrumental Analysis

#### 2.5.1. Determination of Biomass Productivity, Specific Growth Rate, and Chlorophyll a

A total of 10 mL of ALE-*Cv* cultures were taken into 50 mL centrifuge tubes and centrifuged at 4700 rpm for 10 min to separate the supernatant and pellet. The supernatants were discarded, and the pellets were resuspended in 20 mL of deionised distilled water. The centrifugation was then repeated at 4700 rpm for 10 min, and the pellets in the centrifuge tubes were dried at 50 °C overnight or until a constant weight was obtained. The biomass concentration data were used to determine biomass productivity (Bp) (g/L/day) using Equation (1), as shown below.

(1) Bp = (N_7_ − N_0_)/7

where N_7_ and N_0_ are biomass concentrations (g/L) at day 7 (end of the cultivation) and day 0 (start of the cultivation). The specific growth rate (μ) was determined by Equation (2).

(2) μ = (In N_2_ − In N_1_)/(t_2_ − t_1_)

where N_1_ and N_2_ are biomass concentrations (g/L) at day t_1_ (start of the exponential phase) and day t_2_ (end of the exponential phase), respectively, during the exponential phase of growth. All experiments were conducted in triplicate.

The extraction of chlorophyll-a and its quantification were conducted with a slight modification as reported by Hena et al. [27]. A total of 10 mL of ALE-*Cv* culture was centrifuged at 4700 rpm for 10 min. The supernatant was discarded, and the pellet was collected. The pellet was resuspended in 10 mL of methanol: water (9:1; *v*/*v*) and incubated in 20 mL centrifuge tubes for 10 min at room temperature (23 ± 2 °C) to extract chlorophyll-a. The incubated culture was centrifuged again at 4700 rpm for 10 min. The supernatant was collected and designated as S1, and the extraction process was repeated to ensure the complete extraction of chlorophyll-a from the biomass, producing supernatant S2. Both supernatants (S1 + S2) were mixed, and the absorbance of the mixed supernatant was measured at 665 nm and 652 nm. The chlorophyll-a concentration of the extract was calculated using the following Equation (3).

(3) Chlorophyll-a (μg L − 1) = 16.29 × A665 − 8.54 × A652

where A665 and A652 were the absorbance of the supernatant at the wavelengths 665 nm and 652 nm, respectively.

An elemental analyser is used to analyse the content of carbon, hydrogen, and nitrogen in oven-dried biomasses of ALE-*Cv*. The bio-fixation rate of CO_2_ was calculated based on the carbon content data obtained from the elemental analyser using the following Equations (4) and (5).

(4) Bio-fixation rate of carbon (g C/L/day); Z = ((X_c7_ × N_7_) − (X_c0_ × N_0_))/7

(5) Bio-fixation rate of CO_2_ (g/L/day) = Z × MW_CO2_/MW_C_

where Z bio-fixation rate of carbon per day. X_c7_ and X_c0_ represent carbon mass fractions in the biomass samples collected on day 7 and day 0, respectively, and MW represents molecular weight in g/mol.

To determine the ash and volatile matter fractions, the known quantity (1 g) of biomass feedstock was subsequently incubated at 550 °C for 5 h inside the muffle furnace [28]. The ash residue was determined by weighing, while the volatile matter fraction was determined by subtractingthe ash content from the weight of biomass feedstock. The quantification for the ash content was calculated using Equation (6) as below.

(6) Ash content (%) = (Final weight × 100)/Initial weight

#### 2.5.2. Determination of Total Carbohydrates, Protein, and Lipids

Total carbohydrates were quantified with slight modification in Dubois et al. [29] as described by Hena et al. [30]. Approximately 6 mg of measured biomass feedstock was taken into a test tube and hydrolysed with 200 μL of H_2_SO_4_. We incubated the test tube in a water bath at 100 °C for 5 h, then cooled it to room temperature. A total of 9.8 mL of DI water was added into the test tube and centrifuged at 3500 RPM, and the supernatant (hydrolysate) was separated and filtered using a 0.45 μm glass fibre syringe filter. A triplicate of 300 μL of the filtered supernatant was transferred into three separate 2 mL glass tubes, followed by adding 1.25 mL of H_2_SO_4_ and 300 μL of phenol. The content in the test tube was mixed well and incubated at 25–30 °C for 15 min, followed by recording the absorbance at 490 nm using a UV-Vis spectrophotometer (Jasco V-670, Hachioji, Japan). The total carbohydrate was calculated using the calibration curve obtained from the standard glucose solutions.

About 20 mg of biomass feedstock was taken in a 15 mL Falcon tube and added with 10 mL lysis buffer to break the cell structure. The mixture was incubated for 20 min. A 0.1 mL supernatant from the Falcon tube was transferred into a 5 mL glass tube and mixed with 0.1 mL sodium dodecyl sulphate salt (0.05 g/L). Then, 1 mL of alkaline copper reagent was added to the mixture, vortexed, and incubated for 10 min at room temperature. It followed the addition of 0.1 mL of Folin reagent into the mixture, vortexed, and incubated again for 30 min in a dark environment to prevent degradation of the Folin reagent. The absorbance reading was taken at 750 nm using a spectrophotometer. The reference blank was prepared by replacing the protein sample with distilled water. The analysis was carried out in triplicate. Bovine serum albumin (BSA; 1 mg/mL) from Sigma-Aldrich, Auckland, New Zealand, was used as a standard for protein amount determination.

About 1 g biomass feedstock was transferred into 200 mL of a 1:2 chloroform–methanol (*v*/*v*) mixture and homogenised for 10 min. Lipids were extracted in a Soxhlet apparatus operated at 70 °C for 10 h. After Soxhlet extraction, the algal solid residues were removed by passing the suspension through a Whatman GF/C glass fibre filter. The filtrate was carefully collected in a separating funnel containing 2 mL of 1.0% NaCl solution to wash out water-soluble components. The mixture was shaken vigorously for 2 to 3 min and allowed to undergo phase separation. The lower chloroform layer was collected and dried in a rotary evaporator till it attained a constant weight. The total lipid was then quantified gravimetrically [31].

#### 2.5.3. FESEM/EDS Analysis of Precipitate Formed

The chemical composition of the precipitates formed during cultivation was examined using Field Emission Scanning Electron Microscopy coupled with Energy Dispersive X-ray Spectroscopy (FESEM/EDS), MIRA3 TESCAN, Brno, Czech Republic. At the end of the 7-day cultivation period, 100 mL of homogenised culture was vacuum-filtered through a 0.2 µm glass fibre filter to recover the biomass and associated precipitates. The collected residue was dried at 50 °C overnight, or until a constant weight was achieved.

A small portion of the dried sample was coated with a 3–5 nm platinum layer and mounted onto an aluminium stub for imaging. FESEM/EDS analysis was performed at an accelerating voltage of 5 kV, a working distance of approximately 6 mm, using a 20 µm aperture for secondary electron imaging, and a 60 µm aperture for backscattered electron imaging. Elemental maps obtained from the EDS spectra were used to infer the chemical composition of the precipitates.

## 3. Results

Although substantial research has been devoted to reducing the cost of microalgae cultivation, high energy and water demand remain major barriers to large-scale deployment. This study addresses these challenges by enhancing biomass productivity using a CO_2_-adapted strain of *Chlorella vulgaris* capable of growth at 10% CO_2_, outperforming its wild-type counterpart. The primary objective was to investigate the influence of operational parameters on biomass productivity and to determine optimal cultivation conditions.

### 3.1. The Effect of Photoperiods on the Growth of ALE-Cv

**Figure 1:**
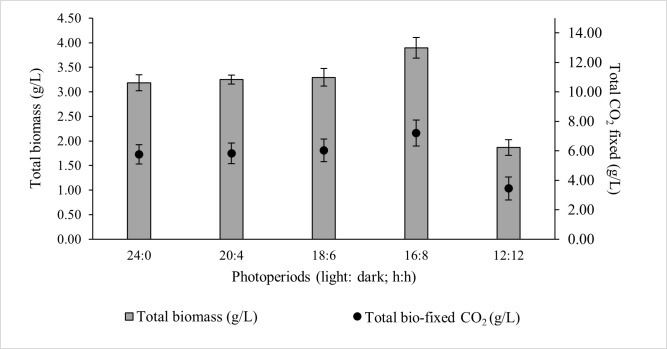
Effect of photoperiod on the total biomass production and total CO_2_ fixed by ALE-*Cv.* The culture was cultivated at 28 °C in a 1 L bioreactor with 0.4 L working volume for 7 days.

Microalgal growth rate is strongly influenced by the efficiency with which light reaches the intracellular photosynthetic pigment chlorophyll-a [32]. The daily light dose determines the amount of glucose produced via photosynthesis, which serves as the primary carbon precursor for metabolic pathways and ultimately governs biomass accumulation, together with the availability of macro- (N, P, K) and micro-nutrients in the growth medium [33]. However, continuous illumination is detrimental, as a dark phase is essential for cellular homeostasis, removal of toxic by-products, and maintenance of circadian regulation of carbohydrate storage and recovery [34]. Among the photoperiods tested, the 16:8 light–dark cycle yielded the highest total biomass concentration (3.894 ± 0.21 g/L) and total CO_2_ bio-fixation (7.202 ± 0.88 g/L). Photoperiods 24:0, 20:4, and 18:6 resulted in comparable biomass and CO_2_ bio-fixation, whereas 12:12 produced the lowest values (Figure 1).

For the optimal 16:8 photoperiod, the biomass productivity, CO_2_ bio-fixation rate, and specific growth rate over seven days were 0.554 ± 0.02 g/L/day, 1.029 ± 0.10 g/L/day, and 0.794 ± 0.07 day^−1^, respectively (Table 2). The rate of CO_2_ fixation showed a strong positive correlation with biomass growth, confirming the direct link between ALE-*Cv* growth and its carbon sequestration capacity. A statistical analysis was conducted to evaluate the significance of photoperiod using one-way ANOVA (Single Factor), and the detailed results are presented in Supplementary Table S1. Photoperiods of 24:0, 20:4, and 18:6 exhibited *p*-values greater than 0.05, indicating no statistically significant effect on biomass productivity. In contrast, photoperiods of 16:8 and 12:12 showed *p*-values below 0.005, demonstrating that biomass production becomes significantly dependent on photoperiod when the light duration is reduced to less than 18 h.

Chlorophyll-a content remained relatively constant up to an 18 h light period; however, it declined at longer light exposures. Biochemical composition showed minor variation across photoperiods: carbohydrate content increased slightly with increasing light duration, protein content decreased, and lipid accumulation was highest at 16:8 and 18:6. Ash content increased with longer light periods, suggesting greater formation of carbonate minerals under prolonged CO_2_ exposure.

### 3.2. The Effect of Light Intensity on the Growth of ALE-Cv

Light intensity directly influences photosynthetic activity in microalgae, although its effective delivery to cells is reduced as it passes through air, vessel surfaces, culture media, and increasingly dense biomass [35]. As cell concentration increases during cultivation, self-shading becomes more pronounced, leading to reduced light penetration through the culture [36]. Therefore, although the cultivation environment is controlled, cell density remains an intrinsic variable affecting light availability.

**Figure 2:**
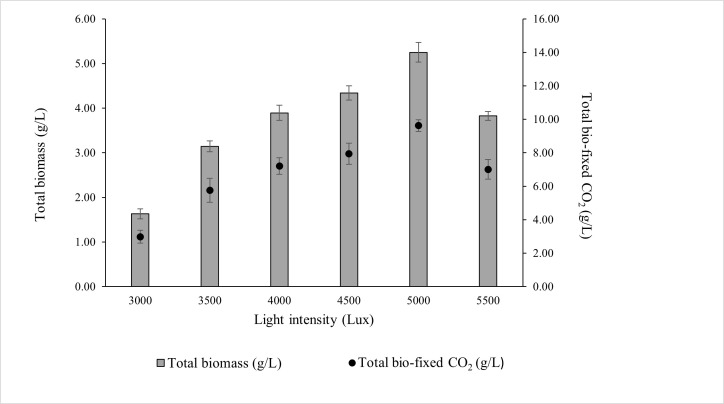
Effect of light intensity on the total biomass production and total CO_2_ fixed by ALE-*Cv.* The culture was cultivated at 28 °C in a 1 L bioreactor with 0.4 L working volume for 7 days under the photoperiod regime of 16:8 h light and dark.

ALE-*Cv* was cultivated under a constant photoperiod of 16:8 h (light:dark) to investigate the effects of different light intensities. As shown in Figure 2, the maximum biomass concentration (5.251 ± 0.22 g/L) and total CO_2_ bio-fixation (9.622 ± 0.35 g/L) were achieved at 5000 lux after 7 days of cultivation. Biomass production and CO_2_ fixation increased by 34.8% and 33.6%, respectively, when light intensity increased from 4000 to 5000 lux.

However, further increases beyond 5000 lux resulted in a decline in biomass accumulation and CO_2_ fixation, likely due to photoinhibition, where excess irradiance causes photo-oxidative stress and reduces photosynthetic efficiency. At the optimal intensity of 5000 lux, the biomass productivity, CO_2_ bio-fixation rate, and specific growth rate were 0.750 ± 0.09 g/L/day, 1.375 ± 0.12 g/L/day, and 0.836 ± 0.11 day^−1^, respectively, as summarised in Table 3.

Light intensity had a pronounced effect on chlorophyll-a content, which increased progressively with increasing light intensity. The highest chlorophyll-a concentration was observed at 5000 lux, corresponding to the highest biomass production. However, further increases in intensity beyond 5000 lux reduced chlorophyll-a content and biomass accumulation, suggesting the onset of photoinhibition.

The biomass-to-chlorophyll-a ratio remained relatively constant across most intensities, except for 3000 lux, where chlorophyll-a concentration was higher than expected relative to biomass. At this low intensity, irradiance was insufficient to support optimal photosynthesis, yet not high enough to induce photodamage, which may explain the elevated chlorophyll-a accumulation as a compensatory response. In contrast, at 5500 lux, both biomass and chlorophyll-a content declined, indicating that excessive light likely caused damage to the photosynthetic apparatus. Under high light intensity, chlorophyll molecules absorb more excitation energy than can be dissipated through photochemical quenching. To avoid the harmful effects of excess, non-utilised energy, singlet chlorophyll undergoes intersystem crossing to form triplet chlorophyll [37]. Carotenoids present in the light-harvesting antenna complex quench triplet chlorophyll, preventing uncontrolled energy transfer to other biomolecules [38]. However, under conditions where triplet chlorophyll accumulates excessively, carotenoids cannot sufficiently dissipate energy. Consequently, triplet chlorophyll reacts with molecular oxygen produced during photosynthesis, leading to the formation of singlet oxygen (^1^O_2_), a common reactive oxygen species (ROS) [39]. Under prolonged photoperiods and high light intensity, the rate of ^1^O_2_ generation exceeds the scavenging capacity of chloroplasts, resulting in damage to photosystem II and a subsequent decrease in photosynthetic capacity and biomass productivity [40].

Light intensity also modulated the biochemical composition of ALE-*Cv* biomass (Table 3). Carbohydrate content decreased with increasing light intensity from 3000 to 5500 lux, while lipid content increased across the same range. Protein levels remained relatively constant regardless of light intensity. Previous studies reported maximum lipid accumulation in *Chlorella vulgaris* and *Nannochloropsis* sp. at approximately 4500 lux [14,41]. In contrast, this study identified 5000 lux as the optimal intensity, which may be attributed to the substantially higher cell density (5.25 g/L) and specific growth rate (0.836 ± 0.11 day^−1^) achieved by ALE-*Cv*, compared with earlier reports of lower biomass (2.05 ± 0.10 g/L) and slower growth (0.339 day^−1^). Based on these findings, 5000 lux was selected as the optimal light intensity for subsequent optimisation experiments.

Additionally, the ash content (Table 3) supports the hypothesis that higher irradiance promotes the formation of inorganic deposits, likely due to enhanced carbonate precipitation under elevated CO_2_ supply and longer exposure to high-intensity light. Natsi and Koutsoukos [42] similarly reported that increased irradiance in microalgal cultures can stimulate inorganic carbonate formation, as elevated CO_2_ uptake and enhanced photosynthetic activity raise local pH and drive carbonate precipitation (e.g., CaCO_3_).

### 3.3. The Effect of pH of the Culture Media on the Growth of ALE-Cv

In photoautotrophic cultivation, microalgae utilise an inorganic carbon source and light energy to synthesise organic compounds for growth [43]. Depending on the species and environmental conditions, microalgae can utilise both dissolved CO_2_ and HCO_3_^−^ as carbon sources. Generally, species adapted to acidic or neutral media preferentially consume dissolved CO_2_, while those adapted to alkaline environments predominantly utilise HCO_3_^−^ [44].

Dissolved CO_2_ enters microalgal cells via passive diffusion, which requires no energy, whereas HCO_3_^−^ transport occurs through active transport mechanisms and is consequently slower. Therefore, pH plays a critical role in determining the dominant mode of inorganic carbon uptake during photosynthesis. CO_2_ dissolves in water to form carbonic acid (Equation (7)), which can further dissociate into bicarbonate and hydrogen ions (Equation (8)):

(7) $$CO_{2}(aq)+H_{2}O⇌H_{2}CO_{3}$$

At acidic-to-neutral pH, the equilibrium of Equation (7) favours the formation of dissolved CO_2_, facilitating its uptake by microalgae [43]. In contrast, at alkaline pH, Equation (8) shifts toward HCO_3_^−^ formation, promoting bicarbonate utilisation.

(8) $$H_{2}CO_{3}(aq)⇌H^{+}+HCO_{3}^{-}$$

**Figure 3:**
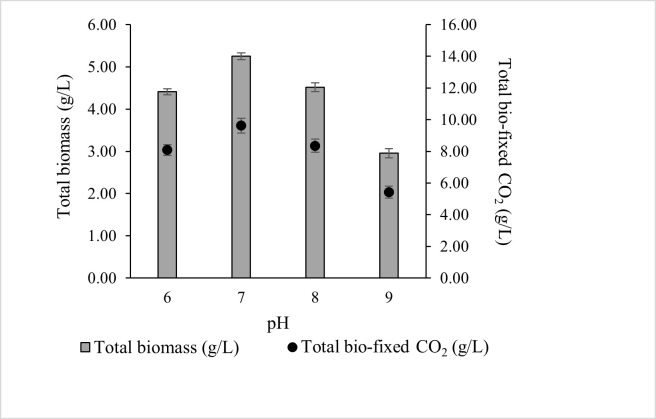
The effect of pH of the culture media on total biomass and total bio-fixed CO_2_. The culture was cultivated at 28 °C in a 1 L bioreactor with 0.4 L working volume for 7 days under 5000 lux intensity and the photoperiod regime of 16:8 h light and dark.

In the present study (Figure 3), the optimal pH for total biomass production was 7 (5.252 ± 0.08 g/L), although comparable productivity was observed at pH 6 (4.411 ± 0.07 g/L) and pH 8 (4.518 ± 0.10 g/L). Biomass growth declined at higher pH, with pH 9 yielding 2.957 ± 0.11 g/L. Correspondingly, total CO_2_ bio-fixation was highest at pH 7 (9.622 ± 0.47 g/L), followed by pH 8, 6, and 9. These results indicate that ALE-*Cv* is capable of utilising both dissolved CO_2_ and HCO_3_^−^, but growth and CO_2_ fixation are maximised near neutral pH.

The total biomass production decreased by 15.9%, 13.9%, and 43.6% at pH 6, 8, and 9, respectively, compared to pH 7. Correspondingly, the CO_2_ capture capacity of ALE-*Cv* declined with deviation from the optimal pH. The reduced growth at basic pH may be attributed to the unavailability of essential elements, which are expected to form carbonates and hydroxides [45]. In contrast, at lower pH, the culture medium contains less HCO_3_^−^, a key inorganic carbon species, limiting microalgal growth. Additionally, under acidic conditions, microalgal cells actively maintain cytoplasmic pH by reducing H^+^ permeability through proton pumps [46]. This energy-consuming process further hinders growth.

For the optimal condition (pH 7), the biomass productivity, CO_2_ bio-fixation rate, and specific growth over seven days were 0.750 ± 0.05 g/L/d, 1.375 ± 0.14 g/L/d, and 0.836 ± 0.09 1/d, respectively, as shown in Table 4. Chlorophyll-a content was proportional to total biomass, and the biomass-to-chlorophyll-a ratio remained constant across the entire pH range studied. The ALE-Cv biomass obtained at pH 6 and 7 exhibited similar biochemical composition, containing comparable amounts of carbohydrates, proteins, and lipids. Notably, protein content remained stable across the full pH range (6–9).

Gaysina [18] reported that at high H^+^ concentrations, the total positive charges on the cell surface increase, disturbing the electrostatic equilibrium. Consequently, cells adapt morphologically by reducing their surface area-to-volume ratio to minimise stress caused by excess H^+^, increasing in cell volume.

**Figure 4:**
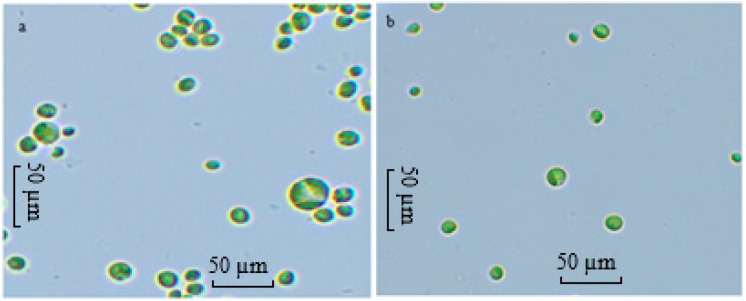
The microscopic image of the cells of ALE-*Cv* cultivated at pH 6 (**a**) and pH 7 (**b**). The slides were prepared with the biomass harvested on day 7.

In the present study, ALE-*Cv* cells cultivated at pH 6 and 7 were compared under a microscope at 40× magnification, and the results are shown in Figure 4a,b. It is evident from Figure 4 that cells grown at pH 6 were larger in size than those cultivated at pH 7. However, not all cells could adapt, which may partly explain the reduced growth observed at lower pH.

**Figure 5:**
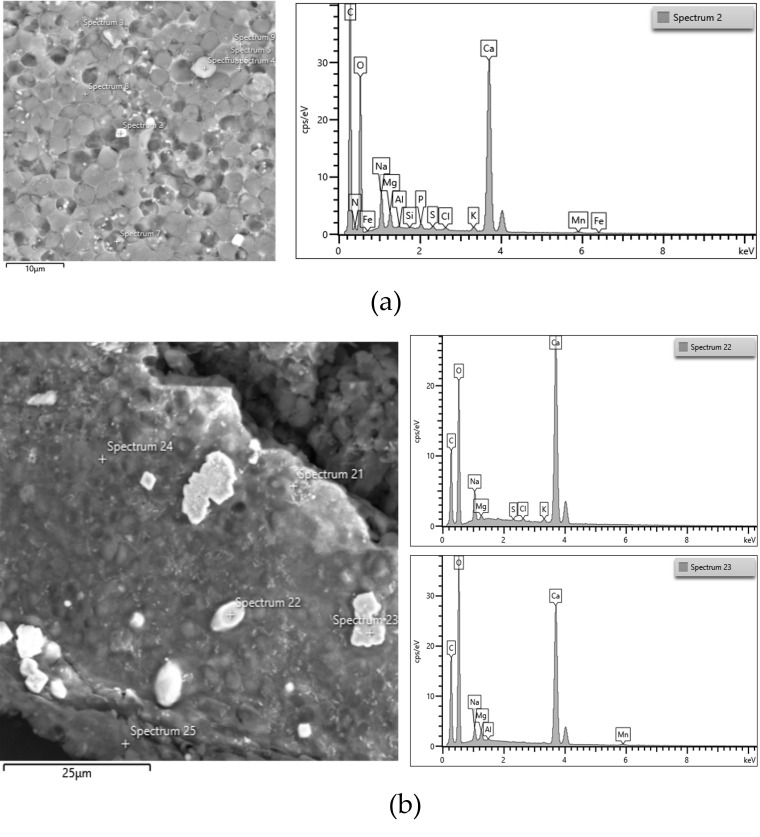
FESEM image, EDS spectrum of the biomass of ALE-*Cv* cultivated at pH 7 (**a**) and pH 9 (**b**). The white crystals are CaCO_3_, as shown in spectra 2, 22, and 23 on the right side.

Ash content was a little higher in biomass cultivated at pH 7 (2.77% of total dry biomass) than at pH 6 (2.55% of total dry biomass). This might be due to the presence of HCO_3_^−^ at pH 7, which formed some carbonate compounds. It was observed that ash content increased with increasing pH from 7 to 9. The FESEM images (Figure 5a,b) further support this finding. As shown in Figure 5a,b, the biomass cultivated at pH 9 contained a higher abundance of CaCO_3_ crystals than that cultivated at pH 7.

### 3.4. The Effect of Inoculum Size on the Growth of ALE-Cv

The inoculum is defined as a culture of ALE-*Cv* grown in EPS-rich and nutrient-poor media. In the present study, such a medium is referred to as an exhausted culture medium. Optimising the ratio of cell concentration to the volume of exhausted medium is critical for effective cultivation. Since the total working volume of the photobioreactor was constant at 400 mL, increasing the inoculum volume correspondingly reduced the available nutrients in the photobioreactor.

Inoculum size directly influences biomass production. A larger inoculum provides more cells for replication, typically resulting in higher biomass. However, excessively large inoculum sizes can induce growth-limiting stress due to rapid nutrient depletion and self-shading effects [9]. Self-shading reduces light penetration, directly affecting photosynthesis and biomass accumulation. Zhang et al. [47] reported that limited light penetration in phototrophic cultures induces stress in microalgal cells, triggering EPS (extracellular polymeric substance) production, which increases media viscosity and adversely affects cellular metabolic activity and growth.

Therefore, selecting an appropriate inoculum size is essential to optimise both biomass growth and CO_2_ fixation. Inoculum size can be adjusted either by varying the inoculum volume while keeping cell concentration constant or by varying the cell concentration within a fixed inoculum volume. In this study, the inoculum size was carefully varied to determine the optimal combination of volume and biomass concentration for maximal growth and CO_2_ fixation of ALE-*Cv*. The range of inoculum volumes and concentrations is provided in Table 1, and a summary of specific inoculum sizes is presented in Table 5. The letters “A,” “B,” and “C” correspond to inoculum volumes of 4 mL, 20 mL, and 40 mL, respectively, while the number following the letter indicates the biomass concentration. For example, A1 refers to 4 mL of ALE-*Cv* culture at a biomass concentration of 0.1 g/L.

**Figure 6:**
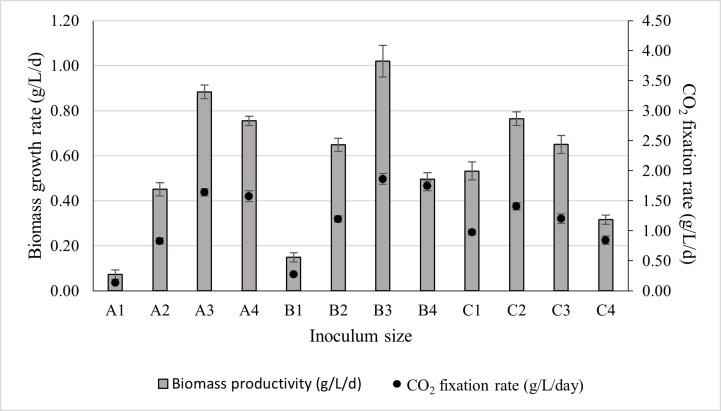
The effect of inoculum size on biomass productivity and its corresponding CO_2_ fixation rate. The culture was cultivated at 28 °C in a 1 L bioreactor with 0.4 L working volume for 7 days under 5000 lux intensity and the photoperiod regime of 16:8 h light and dark.

Since the inoculum sizes varied across experimental runs, biomass productivity was considered a more reliable metric for identifying the optimal cultivation condition. The biomass productivity obtained for different inoculum sizes, along with their corresponding CO_2_ fixation rates, is presented in Figure 6. As shown in Figure 6, inoculum B3 demonstrated the highest biomass productivity, reaching 1.02 ± 0.07 g/L/d, with an associated CO_2_ fixation rate of 1.86 ± 0.09 g/L/d.

Therefore, the inoculum size B3 (20 mL at 10 g/L) was selected for subsequent optimisation experiments. Across all inoculum volumes, biomass productivity increased with rising biomass concentration up to an optimal point, after which it began to decline. The decline in growth rate became more pronounced at higher inoculum volumes. This trend may be explained by the fact that increasing the inoculum volume reduced the proportion of fresh nutrient-rich medium available in the reactor. At the same time, self-shading effects intensified, further limiting photosynthetic efficiency. Additionally, larger inoculum volumes introduced a greater amount of exhausted medium and extracellular polymeric substances (EPS) carried over from the previous culture, which likely increased medium viscosity and imposed further mass transfer and metabolic constraints.

Conversely, a highly concentrated but small-volume inoculum can impose physiological stress on cells during the early adaptation phase. Li et al. [48] reported that *Chlorella vulgaris* experienced physiological stress when exposed to abrupt changes in environmental conditions. In the present study, inocula A2 and C1 contained comparable biomass amounts; however, the biomass productivity was higher in C1. The likely reason is that A2 contained a smaller fraction of the exhausted medium from the previous culture, whereas C1 retained more of it, allowing a smoother physiological transition and reducing adaptation stress.

As cultivation progressed and cell density increased, nutrient availability became more critical than environmental adaptation stress. This interpretation is supported by the results from inocula A3 and C2, which also contained equivalent biomass. In this case, nutrient limitation became the dominant factor affecting growth, as the photobioreactor inoculated with C2 received fewer nutrients due to a larger inoculum volume. Consequently, the biomass productivity was higher in A3 than in C2. The same trend was observed for inocula A4 and C3, further reinforcing this relationship.

The biomass-to-chlorophyll-*a* ratio remained generally constant across all runs, except for bioreactors A4, B4, C3, and C4, which contained an initial biomass concentration ≥ 1 g/L on day 0. These cultures exhibited a noticeably higher chlorophyll-*a* content. This suggests that a high initial biomass concentration stimulated chlorophyll accumulation in the cells.

Cells in denser cultures are more likely to experience self-shading, where light penetration is reduced due to mutual shading among cells. In response, microalgae can increase their chlorophyll content to maximise light absorption and maintain photosynthetic performance under limited light conditions [49].

The biomolecular composition of ALE-*Cv* biomass harvested on day 7 was generally similar across all inoculum sizes, except in cultures initiated with very low biomass concentrations (A1 and B1). In these treatments, the carbohydrate content was higher, and the lipid content was lower than in biomass obtained from other inoculum sizes. This pattern may be attributed to the surplus availability of nutrients in A1 and B1. When nutrients are abundant, microalgae preferentially channel cellular carbon toward carbohydrate synthesis to support rapid growth. In contrast, under nutrient limitation, microalgae typically experience metabolic stress and convert carbohydrates into storage lipids, resulting in reduced carbohydrate and elevated lipid fractions [50].

Based on the experimental results (Figure 6 and Table 6), it is evident that the individual (simple average) effects of cell concentration and culture volume do not fully capture their influence on biomass production due to the presence of complex interactions between these variables. To quantitatively assess these observations, a statistical analysis was conducted using two-way ANOVA to evaluate the significance of inoculum size by examining different combinations of initial cell concentration and culture volume, with respect to nutrient limitation and self-shading effects on biomass production. A summary of the two-way ANOVA results is provided in Supplementary Table S2. Both main effects, cell concentration and culture volume, exhibited *p*-values below the significance threshold, indicating statistically significant effects on biomass production. Furthermore, the interaction effect between cell concentration and volume was also statistically significant, demonstrating that the effect of cell concentration depends on the culture volume. Consequently, the main effects cannot be interpreted independently, as the influence of one factor varies across the levels of the other.

The ash (inorganic) content remained relatively constant across all inoculum sizes (Table 6), indicating that inoculum size did not play a measurable role in inorganic compound accumulation in the biomass.

### 3.5. The Effect of Aeration on the Growth of ALE-Cv

Aeration, expressed as vessel volumes of gas per volume of culture per minute (VVM), plays a critical role in regulating microalgal growth by enabling efficient gas–liquid mass transfer, particularly for CO_2_ uptake during photosynthesis. Adequate aeration enhances CO_2_ availability, improves nutrient mixing, maintains pH balance, and reduces cell sedimentation and aggregation, thereby promoting better light penetration and supporting higher biomass productivity. However, beyond a certain limit, increasing gas flow does not improve growth due to physiological saturation of the carbon fixation machinery [51].

The maximum CO_2_ fixation rate of microalgae is ultimately governed by (1) the environment (CO_2_ concentration, light intensity, mixing, temperature), and (2) genetics and biochemical constraints on carbon assimilation and storage capacity. Because the cultivation environment was tightly controlled in this study, the aeration rate could be systematically optimised to approach the maximum CO_2_ fixation potential of the ALE-*Cv* strain.

**Figure 7:**
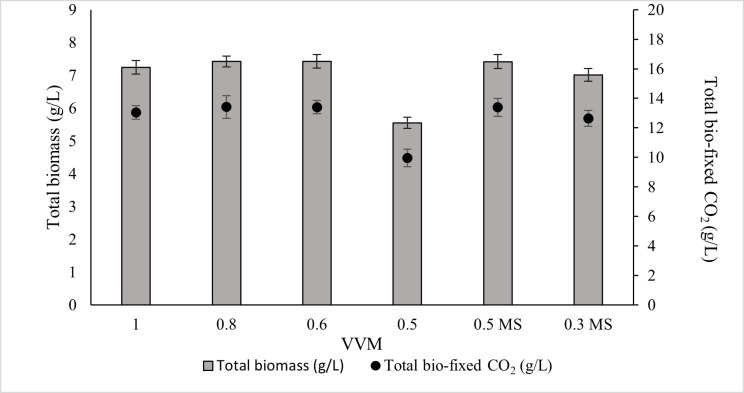
The effect of VVM of supplied gas consisting of 10% CO_2_ mixed with air on total biomass and total bio-fixed CO_2_. 0.5 MS and 0.3 MS are defined as cultures supplied with 0.5 and 0.3 VVM gas (10% CO_2_ mixed with air), aided with a magnetic stirrer (MS) at 100 rpm.

Although an aeration rate of 1.0 VVM is commonly used in laboratory systems, it is not considered economically feasible for large-scale industrial cultivation. Conversely, very low VVM values can substantially reduce biomass productivity. This has led to the question of whether the decline in growth at low VVM results from insufficient CO_2_ supply or from reduced liquid mixing and mass transfer. To confirm this issue, the experiments were conducted with two scenarios: (1) cultures were equipped with different VVM supplies of gas (10% CO_2_ mixed with air) to find the optimal VVM without compromising biomass production or CO_2_ capture capacity in ALE-*Cv*; (2) cultures were aerated below the optimal VVM identified in Scenario 1, but additional mechanical mixing (stirrer agitation) was provided. Growth performance was then compared to determine whether mixing or CO_2_ delivery was the dominant factor limiting productivity at lower gas flow rates. The aeration rate from 1.0 to 0.6 VVM caused no significant change in biomass production or CO_2_ fixation (Figure 7). ANOVA results, as shown in Supplementary Table S3, also showed that the *p*-value for 1 to 0.6 VVM is insignificant. At 0.6 VVM, ALE-*Cv* achieved the highest total biomass yield (7.426 ± 0.21 g/L) and maximum CO_2_ bio-fixation (13.4 ± 0.45 g/L), indicating that 0.6 VVM was the optimal rate under the tested conditions, while at 0.5 VVM, biomass production declined (5.547 ± 0.17 g/L), likely due to insufficient CO_2_ supply and inadequate mixing.

At this flow rate, a noticeable fraction of biomass settled at the bottom of the photobioreactor, suggesting that limited hydrodynamic mixing restricted gas–liquid mass transfer. An extended growth curve of ALE-Cv over 10 days at 0.6 VVM is shown in Figure S1 to evaluate the stationary phase. Numerous studies have previously investigated the effect of aeration rate on biomass productivity. Ronda et al. [52] reported an optimal aeration rate of 1.2 VVM for the growth of *Spirulina platensis*, while Maysitha and Titah [53] identified 4 VVM as optimal for *Chlorella vulgaris* cultivation. In comparison, the optimal aeration rate determined in the present study offers clear advantages, particularly in terms of reduced aeration energy consumption and improved CO_2_ fixation efficiency.

To minimise cell sedimentation at lower aeration rates, a magnetic stirrer was introduced to improve culture homogeneity and enhance CO_2_ dissolution. Therefore, 0.5 VVM and an even lower aeration rate of 0.3 VVM were tested with continuous stirring at 100 rpm. As shown in Figure 7, the combination of 0.5 VVM with magnetic stirring successfully alleviated the mass transfer limitation and supported biomass productivity comparable to that observed at higher aeration rates. In contrast, at 0.3 VVM, the available CO_2_ remained insufficient to sustain growth, and biomass production declined even with mechanical mixing, indicating that CO_2_ limitation became the dominant constraint at this aeration level.

The biomass productivity, CO_2_ bio-fixation rate, and specific growth rate at the optimal aeration of 0.6 VVM were 1.061 ± 0.08 g/L/d, 1.92 ± 0.15 g/L/d, and 0.712 ± 0.04 1/d, respectively (Table 7). No significant change was observed in the chlorophyll-a-to-biomass ratio between 1.0 and 0.6 VVM, indicating that photosynthetic pigment production remained stable under sufficient gas–liquid mass transfer conditions. However, at 0.5 VVM, the chlorophyll-a-to-biomass ratio remarkably decreased, likely due to biomass settling and reduced cell division associated with inadequate CO_2_ and nutrient transfer. This limitation would have decreased photosynthetic activity, causing pigment breakdown and lower chlorophyll-a content. When mechanical mixing was incorporated, the culture maintained both improved biomass production and a stable chlorophyll-a-to-biomass ratio, demonstrating that enhanced mixing can partially compensate for reduced aeration. The biochemical composition of ALE-*Cv* biomass remained comparable across all aeration rates, suggesting that aeration primarily influenced growth kinetics rather than macromolecular allocation.

### 3.6. Semi-Continuous Harvesting of Adaptive Laboratory-Evolved Chlorella vulgaris (ALE-Cv)

A semi-continuous harvesting strategy was implemented to sustain exponential growth and improve overall cultivation efficiency [23]. Based on the findings presented in Section 3.5, which identified 0.6 VVM as the optimal aeration rate for achieving the highest biomass concentration (7.426 ± 0.21 g/L) and CO_2_ bio-fixation (13.4 ± 0.45 g/L), this aeration rate was selected for subsequent semi-continuous operation. To further reduce nutrient and water consumption, a spent-medium reuse approach was incorporated into the semi-continuous design.

**Figure 8:**
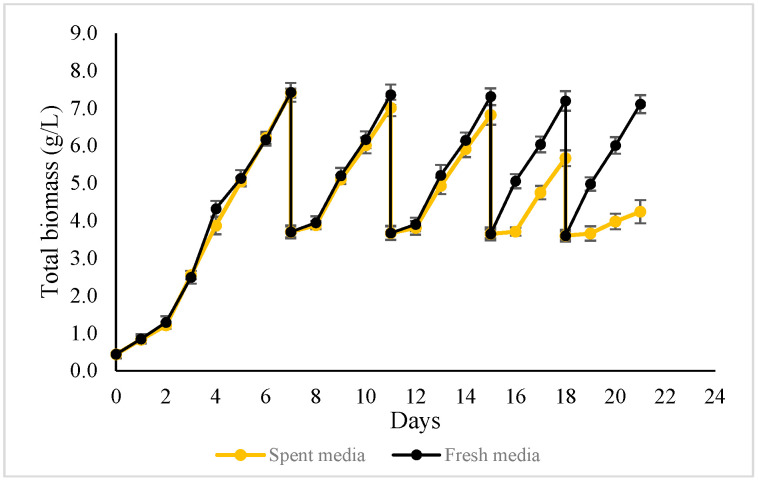
The biomass growth profile of ALE-*Cv* cultivated in semi-continuous harvesting mode, topped up with 50% fresh media (the black curve) and spent media (the yellow curve).

Figure 8 presents the biomass profiles of two semi-continuous harvesting strategies. In both strategies, a freshly prepared medium was used in Cycle 1, followed by four semi-continuous cycles (Cycles 2–5). In the first strategy, approximately 50% of the culture volume was harvested at the end of each cycle, and the same volume of freshly prepared medium was added to restore the working volume. In the second strategy, approximately 50% of the culture was harvested and centrifuged, and the biomass pellet was collected. The supernatant, hereafter referred to as spent medium, was returned to the photobioreactor without replenishing nutrients.

In the first semi-continuous strategy, biomass concentration showed only a marginal decline, from 7.41 g/L in Cycle 1 to 7.11 g/L by Cycle 5, indicating stable and sustained accumulation across cycles. A notable observation was the reduction in cultivation time after Cycle 3, where the duration required to achieve similar biomass concentrations shortened by one day. This improvement coincided with a diminishing lag phase, while a clear lag phase was observed during Cycles 2 and 3; it became negligible from Cycle 4 onward, suggesting enhanced physiological adaptation of ALE-*Cv* to repeated semi-continuous harvesting. Consequently, the average biomass productivity and CO_2_ bio-fixation rate increased over successive cycles. The observed “adaptation” phenomenon during semi-continuous cultivation, characterised by accelerated growth in cycles 4–5, is a notable observation. Future studies will focus on elucidating the underlying physiological mechanisms, such as changes in gene expression and metabolic pathways.

In contrast, in the second semi-continuous strategy, biomass concentration progressively declined with each cycle when spent medium was reused without nutrient supplementation. This indicates progressive nutrient depletion and demonstrates that complete medium recycling without replenishment is not sustainable over extended cultivation.

Semi-continuous harvesting process supplemented with fresh medium initially exhibited slightly lower biomass growth and CO_2_ bio-fixation rates during the 2nd and 3rd cycles at 0.6 VVM aeration (Table 8). However, both parameters gradually increased in the 4th and 5th cycles, indicating physiological adaptation of ALE-*Cv* to repeated semi-continuous harvesting. The chlorophyll-a-to-biomass ratio and biomolecule composition remained consistent across all cycles.

In contrast, the semi-continuous process using spent medium exhibited a distinct trend. Both biomass growth and CO_2_ fixation rates steadily declined over successive cycles, reflecting progressive nutrient depletion. A reduction in the chlorophyll-a-to-biomass ratio was also observed, and the biochemical composition of the biomass showed slight but consistent changes; lipid content increased marginally, while carbohydrate and protein contents decreased. These shifts may indicate the onset of nutritional stress and metabolic reallocation within the cells. Nevertheless, the spent-medium strategy remained effective for up to three cycles without significant compromise to biomass production or CO_2_ capture performance. Inorganic compound (ash) content increased, likely due to the accumulation of extracellular polymeric substances (EPS). Ciempiel et al. [54] reported that the carboxyl, hydroxyl, and carbonyl groups of EPS can interact with ions and facilitate the formation of inorganic compounds.

Although the semi-continuous process supplemented with fresh medium overall bio-fixed the highest CO_2_, demonstrating its superiority over both batch and semi-continuous processes using spent medium in terms of CO_2_ fixation. However, a semi-continuous process supplemented with fresh medium required significantly larger volumes of culture medium. In contrast, the semi-continuous process with spent medium used one-third volume of the semi-continuous process supplemented with fresh medium. This suggests that, in the case of supplementation with fresh medium, nutrients were not fully utilised, which could lead to eutrophication of receiving water bodies if the residual medium were discharged. Considering climate change mitigation and sustainability, we outlined our approach and compared the results to find the best-case scenario without compromising the key needs of both the industrial and environmental sectors. The evaluation was based on the following criteria: (i) maximising CO_2_ fixation and biomass production; (ii) reducing water requirements for cultivation; (iii) improving nutrient utilisation from the culture medium.

The outcomes from the first three cultivation cycles were compared, since both semi-continuous approaches, using either spent medium or fresh medium, displayed comparable growth patterns during this period. As a result, the experiment was concluded after the third cycle. Below is a comparative summary of the two semi-continuous cultivation strategies with batch cultivation for 1 L of culture medium, regardless of duration.

The data in Table 9 clearly demonstrate that, for a fixed culture medium volume, the semi-continuous process using spent medium outperforms the alternative approaches.

Based on these findings, the research group is now developing a continuous cultivation process to enable sustained high-density biomass production and enhanced CO_2_ capture. This ongoing work is part of an industrial-scale effort to establish a cost-effective and resource-efficient ALE-*Cv* cultivation platform.

### 3.7. Bioeconomic Advantages of Semi-Continuous ALE-Cv Cultivation

The semi-continuous harvesting approach applied to adaptive laboratory-evolved *Chlorella vulgaris* (ALE-*Cv*) offers significant advantages for the advancement of green technologies and the bioeconomy. By converting CO_2_ into biomass, ALE-*Cv* cultivation functions as an effective carbon sink, reducing greenhouse gas concentrations. Furthermore, ALE-*Cv* biomass is rich in protein and lipids, providing a sustainable alternative to land-based protein sources. In addition, high-density ALE-*Cv* cultivation can be integrated into wastewater treatment systems, where it removes excess nutrients that drive eutrophication and biodiversity loss. This dual functionality enhances ecosystem resilience and reduces the occurrence of harmful algal blooms, which pose risks to fisheries and public health. Increasing ALE-*Cv* cell density combined with semi-continuous harvesting therefore represents not merely a technical improvement but a systemic strategy that integrates carbon sequestration, sustainable production, and health security within a circular ecological framework. By improving process efficiency and reducing resource demands, high-density ALE-*Cv* cultivation enables deployment at scales sufficient to achieve meaningful climate impact while delivering co-benefits for ecosystems and food systems.

## 4. Conclusions

This study demonstrates that adaptive laboratory-evolved *Chlorella vulgaris* (ALE-*Cv*) can achieve high biomass productivity and CO_2_ bio-fixation under optimised cultivation conditions. Key parameters, including photoperiod, light intensity, pH, inoculum size, and aeration rate, were systematically evaluated. The optimal conditions were identified as 16:8 h light–dark cycles, 5000 lux, pH 7, 20 mL of 10 g/L inoculum, and 0.6 VVM, which maximised growth rate and carbon capture. Semi-continuous cultivation with fresh media further enhanced biomass yield and CO_2_ fixation rate, while reuse of spent media highlighted nutrient limitations over successive cycles. The maximum biomass production in the semi-continuous harvesting process with and without nutrition added was 5.29 ± 0.09 and 9.91 ± 0.11 g/L, while the total corresponding CO_2_ bio-fixation was 9.70 ± 0.13 and 18.16 ± 0.11 g/L, respectively. The findings underscore the potential of ALE-*Cv* as a scalable, sustainable biotechnological platform for carbon sequestration. Importantly, linking high-density ALE-*Cv* cultivation to green technologies and the bioeconomy highlights its broader relevance, demonstrating that efficient microalgal CO_2_ capture can contribute simultaneously to climate change mitigation, ecosystem resilience, and sustainable food and nutritional security.

## Figures and Tables

**Table 1:** The investigation ranges from the process parameters and a series of optimisation steps.

Run	Photoperiods Light:Dark (h:h)	Light Intensity (Lux)	pH	Inoculum Size and Biomass Concentration (Dry-Weight Biomass)	Gas Flow (VVM)
1	12:12, 16:8, 18:6, 20:4, and 24:0	4000	7 ± 0.05	8 mL of 0.75 g/L	1
2	16:8	3000, 3500, 4000, 4500, 5000 and 5500	7 ± 0.05	8 mL of 0.75 g/L	1
3	16:8	5000	6, 7, 8, and 9 ± 0.05	8 mL of 0.75 g/L	1
4	16:8	5000	7 ± 0.05	4 mL of 0.1–100 g/L	1
				20 mL of 0.1–100 g/L	
				40 mL of 0.1–100 g/L	
5	16:8	5000	7 ± 0.05	20 mL of 10 g/L	1, 0.8, 0.6, and 0.5
6	16:8	5000	7 ± 0.05	20 mL of 10 g/L	0.5 and 0.3 with a magnetic stirrer

**Table 2:** Effect of photoperiods on biomass productivity, CO_2_ fixation rate, specific growth rate, and biochemical composition.

Photoperiods	Biomass Productivity (g/L/d)	CO_2_ Fixation Rate (g/L/d)	Specific Growth Rate (1/d)	Chlorophyll-a (µg/L)	Carbohydrate: Protein: Lipid (% Dry Weight)	Ash (% Dry Weight)
12:12	0.264 ± 0.017	0.492 ± 0.09	0.567 ± 0.09	345.6 ± 24.31	23.1 ± 2.4; 51.4 ± 2.6; 21.3 ± 2.6	2.11
16:8	0.554 ± 0.02	1.029 ± 0.10	0.794 ± 0.07	360.2 ± 20.23	24.2 ± 1.2; 50.3 ± 1.9; 22.8 ± 1.4	2.41
18:6	0.469 ± 0.018	0.862 ± 0.08	0.749 ± 0.08	329.5 ± 17.62	25.6± 2.1; 48.3 ± 1.1; 22.4 ± 1.6	3.23
20:4	0.462 ± 0.03	0.832 ± 0.07	0.600 + 0.07	278.4 ± 21.53	25.8 ± 2.6; 48.1 ± 1.7; 21.9 ± 1.1	3.29
24:0	0.453 ± 0.015	0.822 ± 0.06	0.709 ± 0.06	221.2 ± 26.11	26.4 ± 1.6; 48.2 ± 2.6; 21.6 ± 1.4	3.33

**Table 3:** Effect of light intensity on biomass productivity, CO_2_ fixation rate and specific growth rate.

Light Intensity (Lux)	Biomass Productivity (g/L/d)	CO_2_ Fixation Rate (g/L/d)	Specific Growth Rate (1/d)	Chlorophyll-a (µg/L)	Carbohydrate: Protein: Lipid (% Dry Weight)	Ash (% Dry Weight)
3000	0.231 ± 0.09	0.426 ± 0.07	0.550 ± 0.10	278.5 ± 19.13	26.1 ± 2.4; 50.9 ± 2.1; 19.9 ± 1.1	2.36
3500	0.449 ± 0.07	0.822 ± 0.09	0.669 ± 0.08	310.2 ± 24.21	25.9 ± 1.5; 49.8 ± 1.9; 21.2 ± 0.7	2.39
4000	0.554 ± 0.02	1.029 ± 0.10	0.794 ± 0.07	360.2 ± 20.23	24.2 ± 1.2; 50.3 ± 1.9; 22.8 ± 1.4	2.41
4500	0.620 ± 0.10	1.135 ± 0.11	0.809 + 0.12	435.4 ± 18.18	23.7 ± 1.8; 49.1 ± 1.4; 24.6 ± 1.4	2.55
5000	0.750 ± 0.09	1.375 ± 0.12	0.836 ± 0.11	556.9 ± 21.19	19.3 ± 2.1; 49.9 ± 1.8; 27.3 ± 1.1	2.72
5500	0.547 ± 0.08	1.001 ± 0.06	0.791 ± 0.09	405.7 ± 17.23	16.9 ± 1.5; 49.4 ± 0.7; 28.9 ± 0.9	2.96

**Table 4:** Effect of pH of culture media on biomass productivity, CO_2_ fixation rate, specific growth rate, and composition of biomolecules.

pH of Culture Media	Biomass Productivity (g/L/d)	CO_2_ Fixation Rate (g/L/d)	Specific Growth Rate (1/d)	Chlorophyll-a (µg/L)	Carbohydrate: Protein: Lipid (% Dry Weight)	Ash (% Dry Weight)
6	0.630 ± 0.06	1.150 ± 0.11	0.812 ± 0.04	505.2 ± 21.12	19.1 ± 2.6; 49.3 ± 1.9; 27.9 ± 1.9	2.55
7	0.750 ± 0.05	1.375 ± 0.14	0.836 ± 0.09	559.6 ± 24.21	19.3 ± 2.1; 49.9 ± 1.8; 27.3 ± 1.1	2.77
8	0.645 ± 0.05	1.190 ± 0.12	0.815 ± 0.07	512.7 ± 22.19	21.2 ± 1.6; 49.3 ± 2.1; 24.2 ± 1.6	3.74
9	0.422 ± 0.07	0.770 ± 0.13	0.755 + 0.08	325.2 ± 22.87	21.7 ± 1.8; 49.1 ± 1.4; 21.6 ± 1.2	6.55

**Table 5:** Summary and abbreviation of inoculum size studied.

Serial Number	Concentration (g/L)	Inoculum Volume: 1% (*v*/*v*, 4 mL)	Inoculum Volume: 5% (*v*/*v*, 20 mL)	Inoculum Volume: 10% (*v*/*v*, 40 mL)
1	0.1	A1	B1	C1
2	1.0	A2	B2	C2
3	10	A3	B3	C3
4	100	A4	B4	C4

**Table 6:** The effect of inoculum size on the composition of biomolecules and inorganic compounds.

Inoculum Size	Chlorophyll-a (µg/L)	Carbohydrate: Protein: Lipid (% Dry Weight)	Ash (% Dry Weight)
A1	57.8 ± 10.11	26.7 ± 2.2; 53.7 ± 1.3; 16.1 ± 0.2	2.45
A2	354.74 ± 12.12	20.9 ± 2.3; 49.5 ± 1.9; 26.1 ± 0.1	2.47
A3	691.46 ± 12.17	20.2 ± 1.4; 48.9 ± 1.1; 27.9 ± 0.4	2.41
A4	943.01 ± 13.55	20.1 ± 1.1; 48.3 ± 1.5; 28.2 ± 0.7	2.45
B1	120.04 ± 9.22	26.2 ± 2.3; 52.1 ± 1.7; 18.3 ± 0.5	2.44
B2	513.07 ± 13.22	20.7 ± 2.1; 49.4 ± 1.3; 27.2 ± 0.8	2.43
B3	833.50 ± 17.56	20.4 ± 2.2; 49.5 ± 1.5; 27.6 ± 0.4	2.46
B4	1211.52 ± 10.07	19.9 ± 2.5; 49.3 ± 1.6; 28.3 ± 0.3	2.44
C1	429.41 ± 9.87	20.8 ± 2.7; 50.9 ± 1.3; 26.3 ± 0.5	2.51
C2	609.73 ± 11.67	20.7 ± 1.8; 48.9 ± 1.1; 27.8 ± 0.9	2.52
C3	853.21 ± 14.41	20.2 ± 2.2; 48.4 ± 0.9; 28.1 ± 0.2	2.49
C4	2102.31 ± 19.86	20.2 ± 1.8; 48.2 ± 1.1; 28.4 ± 0.5	2.48

**Table 7:** Effect of aeration applied to culture media on biomass productivity, CO_2_ fixation rate, specific growth rate, and composition of biomolecules.

Flow Rate of Gas (10% CO_2_ + 90% Air)	Biomass Productivity (g/L/d)	CO_2_ Fixation Rate (g/L/d)	Specific Growth Rate (1/d)	Chlorophyll-a (µg/L)	Carbohydrate: Protein: Lipid (% Dry Weight)	Ash (% Dry Weight)
1	1.035 ± 0.09	1.86 ± 0.17	0.651 ± 0.07	839.2 ± 11.13	20.22 ± 2.7; 49.5 ± 2.4; 27.9 ± 0.6	2.45
0.8	1.060 ± 0.08	1.92 ± 0.12	0.702 ± 0.09	849.6 ± 14.12	19.90 ± 1.9; 49.7 ± 2.8; 27.1 ± 1.7	2.40
0.6	1.061 ± 0.09	1.92 ± 0.15	0.712 ± 0.04	852.7 ± 12.17	19.21 ± 1.1; 48.9 ± 2.7; 27.2 ± 1.9	2.47
0.5	0.792 ± 0.11	1.42 ± 0.19	0.345 + 0.05	525.5 ± 20.67	20.27 ± 1.8; 49.1 ± 1.3; 27.6 ± 1.3	2.45
0.5 + MS	1.060 ± 0.14	1.91 ± 0.11	0.695 ± 0.06	850.7 ± 19.78	20.23 ± 1.1; 49.6 ± 1.4; 27.7 ± 1.2	2.49
0.3 + MS	1.002 ± 0.13	1.81 ± 0.16	0.448 ± 0.06	765.2 ± 11.54	18.22 ± 2.2; 51.2 ±2.9; 27.1 ± 2.2	2.33

**Table 8:** The biomass productivity, CO_2_ fixation rate, and composition of biomolecules of ALE-Cv cultivated in semi-continuous modes with fresh media and spent media.

Cycle (Days) Media	Biomass Productivity (g/L/d)	CO_2_ Fixation Rate (g/L/d)	Chlorophyll-a (µg/L)	Carbohydrate: Protein: Lipid (% Dry Weight)	Ash (% Dry Weight)
1 (0–7) FM *	1.00 ± 0.11	1.83 ± 0.15	864.2 ± 10.13	20.21 ± 2.1; 49.2 ± 2.2; 26.1 ± 0.5	2.42
2 (7–11) FM	0.91 ± 0.08	1.68 ± 0.11	852.6 ± 11.11	20.19 ± 2.2; 49.4 ± 2.5; 26.9 ± 0.6	2.44
3 (11–15) FM	0.91 ± 0.07	1.67 ± 0.14	852.8 ± 9.12	20.29 ± 2.1; 49.1 ± 2.1; 27.1 ± 1.1	2.47
4 (15–18) FM	1.18 ± 0.12	2.17 ± 0.12	851.5 ± 12.36	19.82 ± 2.5; 48.6 ± 2.3; 27.3 ± 0.9	2.45
5 (18–21) FM	1.17 ± 0.11	2.15 ± 0.13	848.1 ± 13.17	19.53 ± 1.3; 49.4 ± 2.4; 27.6 ± 1.3	2.42
1 (0–7) FM	1.00 ± 0.13	1.83 ± 0.16	862.5 ± 10.35	20.23 ± 1.2; 49.2 ± 1.9; 26.1 ± 1.2	2.43
2 (7–11) SM **^#^**	0.83 ± 0.11	1.52 ± 0.11	827.2 ± 9.32	20.01 ± 1.9; 48.9 ± 2.8; 27.3 ± 1.0	2.51
3 (11–15) SM	0.79 ± 0.12	1.44 ± 0.13	789.2 ± 11.44	19.81 ± 1.1; 48.2 ± 1.4; 27.9 ± 1.3	2.59
4 (15–18) SM	0.67 ± 0.14	1.24 ± 0.14	589.9 ± 12.23	19.44 ± 2.3; 48.2 ± 2.2; 28.5 ± 2.2	2.62
5 (18–21) SM	0.21 ± 0.10	0.39 ± 0.12	406.2 ± 10.65	19.01 ± 1.8; 47.2 ± 1.5; 29.1 ± 1.7	2.72

FM * = fresh media; SM **^#^** = spent media.

**Table 9:** Comparison of semi-continuous scenarios with batch cultivation for 1 L culture media.

Key Metric	Semi-Continuous Process Using Spent Medium *	Semi-Continuous Process Using Fresh Medium *	Batch Cultivation ⁑
Total CO_2_ fixed (g)	18.16	9.70	12.81
Total biomass produced (g)	9.91	5.29	7.03

Over 3 cycles, which was equivalent to 15 days *; batch cultivation was run for 7 days ⁑ .

## Data Availability

The data that support the findings of this study are included in the article/Supplementary Materials. Further inquiries can be directed to the corresponding author.

## Supplementary Materials

The following supporting information can be downloaded at https://www.mdpi.com/article/10.3390/microorganisms14020324/s1. Table S1: Results on the effect of photoperiod on the total biomass production and summaries of the One-Way Analysis of Variance (ANOVA) interpretation.; Table S2: Results on the effect of inoculum size on biomass productivity and a summary of the Two-Way Analysis of Variance (ANOVA) interpretation; Table S3: Results on the effect of gas flow rate on the total biomass production and summaries of the One-Way Analysis of Variance (ANOVA) interpretation; Figure S1: Growth curve of ALE-*Cv* for 10 days at 0.6 VVM.
