# Supplementary material for: Parametric Studies and Semi-Continuous Harvesting Strategies for Enhancing CO2 Bio-Fixation Rate and High-Density Biomass Production Using Adaptive Laboratory-Evolved Chlorella vulgaris
Source: Microorganisms. 2026 Jan 30;14(2):324. doi: 10.3390/microorganisms14020324 (PMC12943192; doi:10.3390/microorganisms14020324)
Supplement: Supplementary file 1 [file microorganisms-14-00324-s001.zip › microorganisms-4077494-supplementary.pdf]

Supplementary Table S1. Results on the effect of photoperiod on the total biomass production and summaries of the One-Way Analysis of Variance (ANOVA) interpretation.

Triplicate of total biomass production at different photoperiods

	Biomass g/L				
Photoperiods (Light: Dark)	24:0	20:4	18:6	16:8	12:12
Set 1	3.184	3.24	3.296	3.894	1.926
Set 2	3.022	3.169	3.116	3.688	1.682
Set 3	3.346	3.348	3.476	4.1	1.99
Mean	3.184	3.25	3.296	3.894	1.866
Standard deviation	0.16	0.090	0.18	0.21	0.16

Summary of ANOVA interpretation for photoperiods 24:0 and 20:4 h

Anova: Single Factor (One-Way)

SUMMARY

Groups	*Count*	*Sum*	*Average*	*Variance*
Column 1 (24:0)	3	9.552	3.184	0.026244
Column 2 (20:4)	3	9.757	3.252333	0.008124

ANOVA

Source of Variation	*SS*	*df*	*MS*	*F*	*P-value*	*F crit*
Between Groups	0.007004167	1	0.007004	0.407594	0.557914	7.708647
Within Groups	0.068736667	4	0.017184			
Total	0.075740833	5				

Summary of ANOVA interpretation for photoperiods 24:0, 20:4 and 18:6 h

Anova: Single Factor (One-Way)

SUMMARY

Groups	*Count*	*Sum*	*Average*	*Variance*
Column 1 (24:0)	3	9.552	3.184	0.026244
Column 2 (20:4)	3	9.757	3.252333	0.008124
Column 3 (18:6)	3	9.888	3.296	0.0324

ANOVA

Source of Variation	*SS*	*df*	*MS*	*F*	*P-value*	*F crit*
Between Groups	0.019120222	2	0.00956	0.42955	0.669349	5.143253
Within Groups	0.133536667	6	0.022256			
Total	0.152656889	8				

Summary of ANOVA interpretation for photoperiods 24:0, 20:4, 18:6 and 16:8 h

Anova: Single Factor (One-Way)

SUMMARY

Groups	*Count*	*Sum*	*Average*	*Variance*
Column 1 (24:0)	3	9.552	3.184	0.026244
Column 2 (20:4)	3	9.757	3.252333	0.008124
Column 3 (18:6)	3	9.888	3.296	0.0324
Column 4 (16:8)	3	11.682	3.894	0.042436

ANOVA

Source of Variation	*SS*	*df*	*MS*	*F*	*P-value*	*F crit*
Between Groups	0.96942025	3	0.32314	11.83616	0.002597	4.066181
Within Groups	0.218408667	8	0.027301			
Total	1.187828917	11				

Summary of ANOVA interpretation for photoperiods 24:0, 20:4, 18:6, 16:8 and 12:12 h

Anova: Single Factor (One-Way)

SUMMARY

Groups	*Count*	*Sum*	*Average*	*Variance*
Column 1	3	9.552	3.184	0.026244
Column 2	3	9.757	3.252333	0.008124
Column 3	3	9.888	3.296	0.0324
Column 4	3	11.682	3.894	0.042436
Column 5	3	5.598	1.866	0.026416

ANOVA

Source of Variation	*SS*	*df*	*MS*	*F*	*P-value*	*F crit*
Between Groups	6.665573067	4	1.666393	61.43597	5.31E-07	3.47805
Within Groups	0.271240667	10	0.027124			
Total	6.936813733	14				

Supplementary Table S2. Results on the effect of inoculum size on biomass productivity and a summary of the Two-Way Analysis of Variance (ANOVA) interpretation.

Triplicate of biomass productivity at different inoculum sizes.

		Volume		
		Group A	Group B	Group C
Concentration	Group 1	0.071	0.15	0.53
		0.068	0.145	0.531
		0.072	0.151	0.529
	Group 2	0.45	0.65	0.76
		0.46	0.651	0.761
		0.44	0.648	0.758
	Group 3	0.88	1.02	0.65
		0.9	1.03	0.651
		0.87	1.01	0.648
	Group 4	0.86	0.52	0.32
		0.75	0.51	0.311
		0.67	0.479	0.328

Summary of the Two-Way Analysis of Variance (ANOVA) interpretation.

Anova: Two-Factor (Two-Way) with replication

SUMMARY	Group A	Group B	Group C	Total
Group 1				
Count	3	3	3	9
Sum	0.211	0.446	1.59	2.247
Average	0.070333	0.148667	0.53	0.249667
Variance	4.33E-06	1.03E-05	0.000001	0.04536
Group 2				
Count	3	3	3	9
Sum	1.35	1.949	2.279	5.578
Average	0.45	0.649667	0.759667	0.619778
Variance	0.0001	2.33E-06	2.33E-06	0.018509
Group 3				
Count	3	3	3	9
Sum	2.65	3.06	1.949	7.659
Average	0.883333	1.02	0.649667	0.851
Variance	0.000233	0.0001	2.33E-06	0.026387
Group 4				
Count	3	3	3	9
Sum	2.28	1.509	0.959	4.748
Average	0.76	0.503	0.319667	0.527556
Variance	0.0091	0.000457	7.23E-05	0.039102
Total				
Count	12	12	12	
Sum	6.491	6.964	6.777	
Average	0.540917	0.580333	0.56475	
Variance	0.109433	0.106585	0.029053	

ANOVA

Source of Variation	*SS*	*df*	*MS*	*F*	*P-value*	*F crit*
Sample	1.67038	3	0.556793	662.4988	3.33542E-23	3.008787
Columns	0.009458	2	0.004729	5.626884	0.00990988	3.402826
Interaction	1.005225	6	0.167537	199.3439	2.9449E-19	2.508189
Within	0.020171	24	0.00084			
Total	2.705234	35				

Supplementary Table S3. Results on the effect of gas flow rate on the total biomass production and summaries of the One-Way Analysis of Variance (ANOVA) interpretation.

Triplicate of total biomass production at different flow rates

	1	0.8	0.6	0.5	0.5MS	0.3MS
Set 1	7.242	7.421	7.436	5.549	7.419	7.023
Set 2	7.132	7.311	7.328	5.523	7.449	6.901
Set 3	7.352	7.531	7.499	5.569	7.389	7.112

Summary of ANOVA interpretation for flow rates 1 and 0.8 VVM

Anova: Single Factor

SUMMARY

Groups	*Count*	*Sum*	*Average*	*Variance*
Column 1	3	21.726	7.242	0.0121
Column 2	3	22.263	7.421	0.0121

ANOVA

Source of Variation	*SS*	*df*	*MS*	*F*	*P-value*	*F crit*
Between Groups	0.048062	1	0.048062	3.972025	0.117049	7.708647
Within Groups	0.0484	4	0.0121			
Total	0.096462	5				

Summary of ANOVA interpretation for flow rates 1, 0.8 and 0.6 VVM

Anova: Single Factor

SUMMARY

Groups	Count	Sum	Average	Variance
Column 1	3	21.726	7.242	0.0121
Column 2	3	22.263	7.421	0.0121
Column 3	3	22.263	7.421	0.007479

ANOVA

Source of Variation	SS	df	MS	F	P-value	F crit
Between Groups	0.064082	2	0.032041	3.034281	0.122882	5.143253
Within Groups	0.063358	6	0.01056			
Total	0.12744	8				

Summary of ANOVA interpretation for flow rates 1, 0.8, 0.6 and 0.5 VVM

Anova: Single Factor

SUMMARY

Groups	Count	Sum	Average	Variance
Column 1	3	21.726	7.242	0.0121
Column 2	3	22.263	7.421	0.0121
Column 3	3	22.263	7.421	0.007479
Column 4	3	16.641	5.547	0.000532

ANOVA

Source of Variation	SS	df	MS	F	P-value	F crit
Between Groups	7.470644	3	2.490215	309.2378	1.31E-08	4.066181
Within Groups	0.064422	8	0.008053			
Total	7.535066	11				

Summary of ANOVA interpretation for flow rates 1, 0.8, 0.6, 0.5 and 0.5 MS VVM

Anova: Single Factor

SUMMARY

Groups	*Count*	*Sum*	*Average*	*Variance*
Column 1	3	21.726	7.242	0.0121
Column 2	3	22.263	7.421	0.0121
Column 3	3	22.263	7.421	0.007479
Column 4	3	16.641	5.547	0.000532
Column 5	3	22.257	7.419	0.0009

ANOVA

Source of Variation	*SS*	*df*	*MS*	*F*	*P-value*	*F crit*
Between Groups	8.097948	4	2.024487	305.7122	2.09E-10	3.47805
Within Groups	0.066222	10	0.006622			
Total	8.16417	14				

Summary of ANOVA interpretation for flow rates 1, 0.8, 0.6, 0.5, 0.5 MS and 0.3 MS VVM

Anova: Single Factor

SUMMARY

Groups	*Count*	*Sum*	*Average*	*Variance*
Column 1	3	21.726	7.242	0.0121
Column 2	3	22.263	7.421	0.0121
Column 3	3	22.263	7.421	0.007479
Column 4	3	16.641	5.547	0.000532
Column 5	3	22.257	7.419	0.0009
Column 6	3	21.036	7.012	0.011221

ANOVA

Source of Variation	*SS*	*df*	*MS*	*F*	*P-value*	*F crit*
Between Groups	8.097958	5	1.619592	219.1994	2.33E-11	3.105875
Within Groups	0.088664	12	0.007389			
Total	8.186622	17				

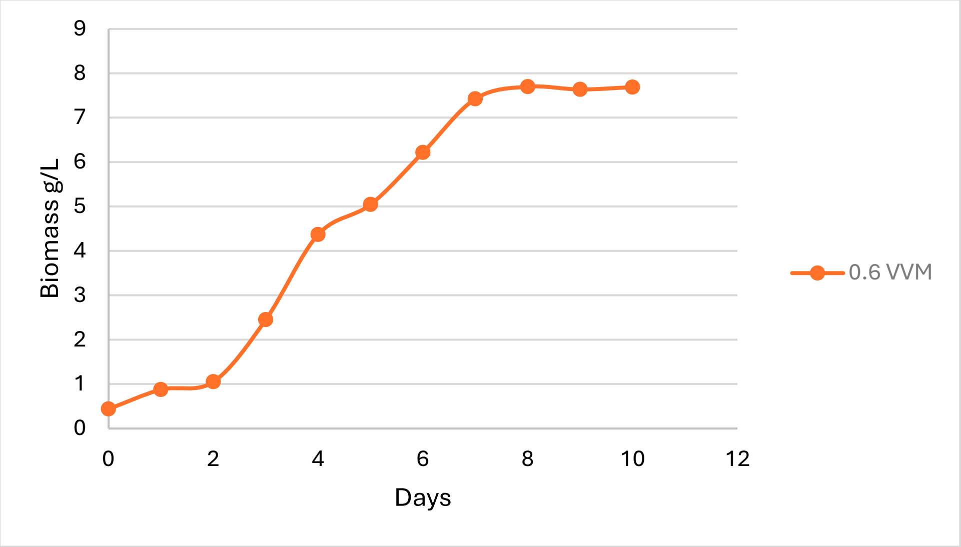

Supplementary Figure S1: Growth curve for 10 days at 0.6 VVM.
